# Feasibility study of volumetric modulated arc therapy for the treatment of retroperitoneal sarcomas

**DOI:** 10.1186/1748-717X-5-83

**Published:** 2010-09-20

**Authors:** Carmen Llacer-Moscardo, François Quenet, David Azria, Pascal Fenoglietto

**Affiliations:** 1 Department of Radiation Oncology, CRLC Val D'Aurelle Paul-Lamarque, Montpellier, France; 2 Department of Surgical Oncology, CRLC Val D'Aurelle Paul-Lamarque, Montpellier, France

## Abstract

**Background:**

Radiotherapy for retroperitoneal sarcomas remains controversial and a technical challenge considering the threshold of contiguous critical organs tolerance. We performed consecutive RapidArc dosimetric plans in preoperative or postoperative setting.

**Methods:**

A dosimetric study was carried out from six preoperative (group A) and four postoperative (group B) CT-scans, performed in 7 patients.

Prescribed dose was 45 and 50 Gy for groups A and B, respectively. The planning target volume (PTV) was defined as the clinical target volume (CTV) plus 5 mm. The CTV encompassed the gross tumor volume (GTV) plus 10 mm or the tumoral bed. The dosimetric plans were optimized on a RapidArc Eclipse console using the progressive resolution algorithm, PRO version 8.8. Normalization method allowed the coverage of 99% of the PTV by 95% of the dose.

**Results:**

Mean PTV were 2318.5 ± 2223.9 cc [range 348-6198 cc] and 698.3 ± 216.6 cc [range 463 -933 cc] for groups A and B, respectively. Plans were optimized for single arcs in group B and for single or two arcs in group A. The contralateral kidney volume receiving 5 Gy (V_5Gy_) was 21.5 ± 23.3% [range 0-55%] and 3.1 ± 2.6% [range 0-7.3%] for groups A and B, respectively. The mean dose received by 1% of the kidney (D_1%_) was 5.6 ± 2.4 Gy [range 3.6 -7.6 Gy] for group A and 5.4 ± 0.7 Gy [range 4.3-6 Gy] for group B. The volume of small bowel excluding the PTV (small bowel-PTV) that received 40 Gy and 30 Gy (V_40Gy _and V_30Gy_) in group A were 7.5 ± 4.4% [range 5.4-14.1%] and 18.5 ± 7.1% [range 10-30.4%], respectively.

In group B, small bowel-PTV V_40Gy _and V_30Gy _were 4.7 ± 3.3% [range 3.3-8%] and 21.6 ± 7.5% [range 9.4-30%] respectively. In a second step, we treated two patients in the postoperative group. Treatment time delivery with one arc was 74 seconds. No severe acute toxicity was observed.

**Conclusion:**

RapidArc technology for retroperitoneal sarcomas showed acceptable dosimetric results in preoperative or postoperative clinical situation. From the first treated patients, acute tolerability was good to excellent.

## Background

Retroperitoneal sarcoma is a rare and very heterogeneous disease representing about 10-15% of all soft tissue sarcomas. Surgery is the main treatment, but microscopic or gross residual disease may remain after the procedure, compromising local control and survival [1-4]. Since local progression rather than metastatic dissemination is the main cause of death, the role of radiotherapy in association to surgery has been investigated. There are no randomized trials comparing postoperative to preoperative radiotherapy and the appropriate strategy is not well defined today.

Based on the results of phase III randomized trials for limb soft tissue sarcoma, postoperative RT has been adopted by some teams in retroperitoneal sarcomas. Nevertheless, this approach raises the problem of the tumor underdosing due to the nearby critical organs at risk (OAR), with the consequence to increase the risk of local recurrence. This concern was confirmed by several authors who reported a high local relapse rate inside the radiotherapy field with considerable toxicity, dissuading postoperative radiotherapy [4-6].

The single randomized trial about adjuvant radiotherapy in resectable retroperitoneal sarcomas [7,8] compared a standard external beam radiotherapy (EBRT) delivering 50-55 Gy to an experimental therapy that associated a single dose (20 Gy) of intraoperative radiotherapy (IORT) using electrons with a low dose postoperative EBRT (35-40 Gy). With a median follow-up of 8 years, the number of locoregional recurrence was significantly reduced in the experimental arm, as well as the enteral toxicity.

Preoperative radiotherapy has some theoretical advantages in the management of retroperitoneal sarcomas, such as the reduction of tumor seeding during surgery and the shift of radiosensitive viscera outside the treatment field [9]. Prospective trials showed the feasibility of preoperative radiotherapy in this context [10-12].

Regarding IMRT, it is now well established that this technique usually provides high conformity and offers improved OAR sparing when compared to 3 D conformational radiotherapy. IMRT use has already been investigated for the treatment of retroperitoneal sarcomas [13-15]. Although large fields may be required for those tumors, more particularly in preoperative setting, this does not preclude the employment of IMRT [14], but the dose inhomogeneity within the target can increase considerably, especially in the vicinity of kidneys. To improve dose homogeneity throughout the planning tumor volume (PTV), multiplying fields may be necessary, having the effect to increase the treatment time per fraction [16]. Some authors investigated the feasibility of diminishing the size of fields to only irradiate specifically the portion of the clinical tumor volume (CTV) at the higher risk of relapse [13].

In this context, the purpose of this study was to assess dosimetric aspects using RapidArc technology for the treatment of retroperitoneal sarcoma. The feasibility of volumetric arc therapy was evaluated in several dosimetric plans obtained before or after surgery. We used two different dose levels (45 and 50 Gy) adapted to the clinical situation, in order to protect normal tissues including small bowel, contralateral kidney and spinal cord and achieve an excellent coverage of the whole target volume. In addition, we investigated the opportunity to deliver complex radiotherapy treatments in a short treatment time. Finally, we directly implemented these physical data into the clinic.

## Methods

This dosimetric study was carried out from ten CT-scans performed in a series of seven consecutive patients with resectable retroperitoneal sarcoma. Patients underwent either a single preoperative or postoperative CT-scan or both exams, providing six preoperative (group A) and four postoperative cases (group B). The dosimetric analysis was performed using RapidArc technology.

### Radiotherapy treatment planning

Patients underwent CT scan-based virtual simulation (GE lightspeed RT16 Milwaukee, USA). Patients were placed in supine position with the arms above the head, using a special support (Sinmed, The Netherlands) and knees were placed with a knee support (Sinmed, The Netherlands). Intravenous contrast was not used considering that renal function of those patients could be altered. 4DCT Scanner was performed to include tumor motion during breathing with 2.5 mm thick slices at 2.5 mm intervals. Tumor (GTV) or tumor bed were manually contoured on the CT images. The isocenter was set in the middle of the GTV if preoperatively or the tumoral bed if postoperatory, using our virtual simulation console (Advantagesim, GE Milwaukee, USA). In the case of preoperative radiotherapy (Group A), the CTV included the tumor and a margin around obtained by a three-dimensional 10 mm expansion, except posteriorly in regards of the vertebral body or bone, where the margin was adapted to sculpt these structures. In the postoperative planning (Group B), the CTV was defined together by the surgeon and the radiation oncologist to include the tumor bed and all the areas at risk. To account for set-up inaccuracies, a PTV was defined by a three-dimensional 5 mm expansion of CTV in all directions, except close to the spinal cord where it was reduced if necessary. The PTV margin was chosen after 4DCT scanner evaluation.

Kidneys or contralateral kidney were completely contoured. A planning organ at risk volume (PRV) of 3 cm was added to the contralateral kidney for two reasons: first, because of the potential internal movement of this structure and second, to be able to define a constraint limiting the dose delivered around the kidney. Small bowel and spinal cord were contoured from 2 cm above to 2 cm below the extension of the tumor or the tumor bed corresponding to the portion of the irradiated organ. Liver was contoured as a whole organ when it was close to the target volume.

The dose prescribed to the PTV was 50 and 45 Gy in 25 fractions for Groups A and B, respectively.

Dose constraints to the OAR were based on the available IMRT studies (Table 1). The maximal dose (D_max_) allowed for the small bowel was the prescribed dose. Dose received by 50% and 30% of the small bowel (SB D_50, _SB D_30_) should not exceed 30 Gy and 40 Gy, respectively. The maximal dose allowed to contralateral kidney was 12 Gy, but we systematically tried to minimize global dose to the whole volume. Liver could receive 20 Gy to the whole volume and 40 Gy to 30% of the volume. The maximal tolerated dose to the spinal cord was 45 Gy.

**Table 1:** Literature dose constrains for IMRT.

Author	n° cases	preop	postop	dose to PTV (Gy)	dose constraints (Gy)			
					**contralateral kidney**	**small bowel**	**spinal cord**	**liver**
					
Tzeng [26]	16	16	0	45 ± 12,5	< 23	< 45	< 45	< 33
						54 to <20 cc		

								
Bossi [13]	18	18	0	50	< 10 to 50%	V55Gy < 50%	< 48	V50Gy < 33%
					< 50	V30Gy < 100%		

								
Koshy [14]	11	9	2	45-50,4	12 to 100%	< 45		
					15 to 50%	D75% 48		V40Gy < 50%
						D50% 50		V30Gy < 100%

Present study	10	6	4	45-50	< 12	< 45-50	< 45	
						V40Gy <30%		V40Gy <30%
						V30Gy <50%		V30Gy <40%

The RapidArc plan optimization was generated by the progressive resolution optimizer (PRO) algorithm of the Eclipse workstation (Varian Medical System, Palo Alto, USA) in a version 8.8 allowing multiple arcs. Single or double gantry rotation was used depending on the thickness of the volume. Each arc had systematically an counter-clockwise rotation of 358° from 179° to 181° and opposite if two arcs. The beams shared the same isocenter with different collimator rotation to increase the modulation capacities of the algorithm.

Plan acceptance criteria required that at least 95% of the dose covers 99% of the PTV volume.

### Evaluation tools

Dose Volume Histograms (DVH) were generated to evaluate the three different plans. For PTV, the parameters D_1% _and D_99% _were used as surrogate markers for maximum and minimum doses. Mean dose (D_mean_) was also reported.

The degree of conformity of the plans was defined as the ratio between the volume receiving at least 95% of the prescribed dose and the volume of the PTV (CI_95%_).

The homogeneity index (HI) was expressed by D_5% _- D_95% _(difference between the dose covering 5% and 95% of the PTV). For all patients DVH for OAR (bowel, bowel excluding PTV, kidneys and spinal cord) were calculated and reported. A set of V_x _values and D_mean _was therefore reported. The number of Monitor Units (MU) per fraction required for each plan and the treatment delivery time (from start to the end of the irradiation), dimension of the fields and collimator angle are reported in Table 2.

**Table 2:** Technical data for RapidArc

Case	Preoperative 1	Preoperative 2	Preoperative 3	Preoperative 4	Preoperative 5	Preoperative 6	Postoperative 1	Postoperative 2	Postoperative 3	Postoperative 4
Dose [Gy]	50	50	50	50	50	50	45	45	45	45

Volume PTV [cc]	6198	384	2535	348	4085	361	895	463	502	933

Lenght PTV [cm]	24	14	22	9,3	31	9,41	29	21	16	24

Number of arcs	2	1	2	1	2	1	1	1	1	1

MU	177+190	337	233+217	433	158+132	372	295	322	374	338

										

Energy [MV]	18	18	6	6	18	18	18	18	18	18

Collimator Angle	30/330	30	45/315	45	35/335	45	345	30	45	30

X jaw [cm]	23/23,4	12,7	22,4/23	12,2	26,4/27,5	11,8	15	15	17,9	23,9

Y jaw [cm]	33,4/33,4	14,7	23,5/24,4	12	36,5/36,5	11,8	30	22,5	18,5	29,5

Following the results of the study, the two last consecutive patients of group B were treated by receiving 45 Gy.

### Quality assurance for treated patients

We conducted a quality control of the dosimetric plans regarding the 2 patients treated in this study. It consisted in a comparison between the previous dose calculated by the planning system and the actual measured dose delivered by the linac. Two different methods were used. The first one consisted of calculating the plan in a cylindrical phantom of 20 cm diameter and then measuring the dose at the central point of this phantom by an ionisation chamber of 0.125 cc (PTW, Freiburg, Germany). The second method used an amorphous silicon portal imager (AS1000 Varian Medical System, Plo Alto, US) as a detection matrix with a resolution of 0.39 mm/pixel at the machine isocenter. The dose collected was compared to a previous distribution on water using the GlaAs algorithm and the Epiqa software (Epidos, Brtaislavia, Slovakia)[17].

## Results

Technical data are summarized in Table 2. Our cases were characterized by very large target volumes involving wide fields until 36 cm of length. This resulted in a low number of MU delivered (380.7 and 332.3 for Groups A and B, respectively) due to a high output factor of the machine. Postoperative plans were optimized for one arc, and some preoperative plans, specially those with the largest PTV, required 2 arcs. Even in those cases, the number of MU was not significantly increased.

For the treated patients, the treatment time was 74 seconds using one arc. Quality control analysis showed acceptable results with a difference between the calculated and measured doses of 1.2% and 1.7% in the cylindrical phantom. Percentage of points meeting the criteria of 3%-3 mm for the gamma index was 98.3% and 95.7% for both patients.

**Figure 1:**
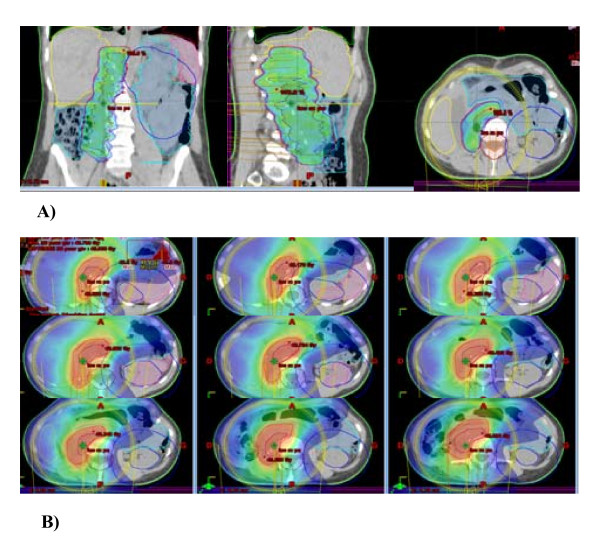
**Conformity of IMRT using RapidArc in a postoperative case**. A) Volume receiving 45 Gy (V45). B) Volume receiving 5 Gy (V5). Contralateral kidney is completely spared.

**Figure 2:**
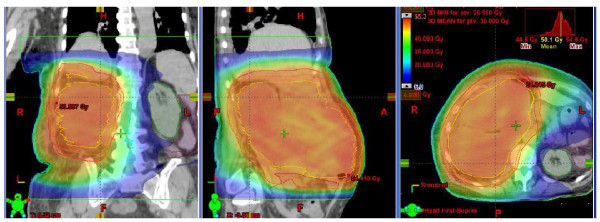
**Conformity of IMRT using RapidArc in a preoperative case**. Dose distribution for a preoperative case. Colourwash is in the interval from 5 to 50 Gy.

**Figure 3:**
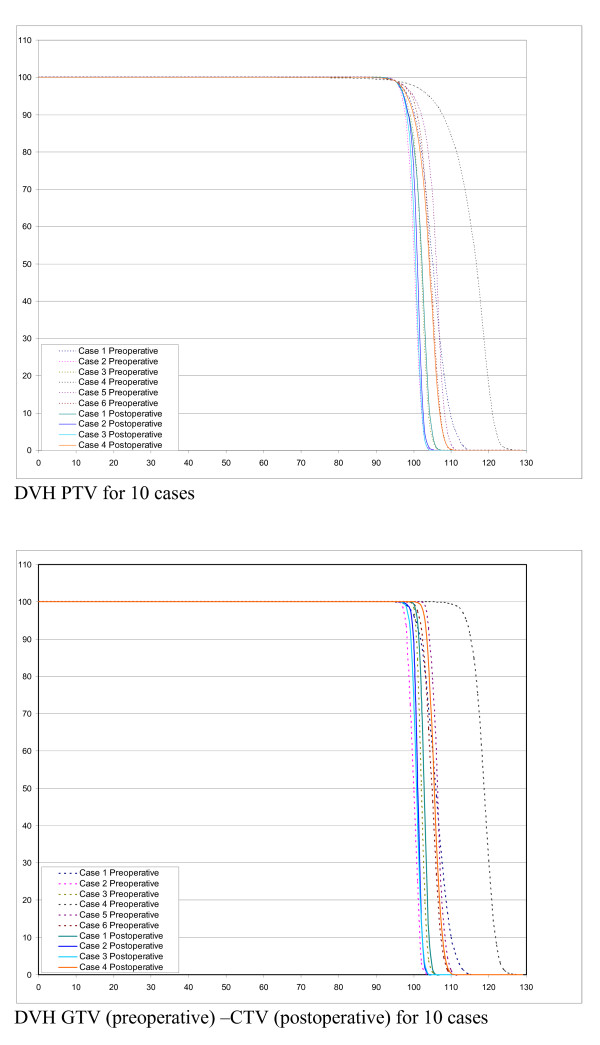
**Dose Volume Histograms for PTV (all cases), CTV(postoperative cases) and GTV (preoperative cases)**.

**Figure 4:**
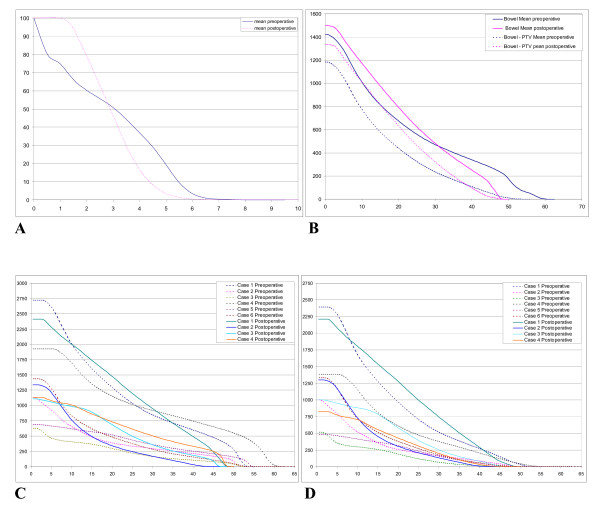
**Dose Volume Histograms (DVH) for OAR**. A) mean DVH for contralateral kidney. B) mean DVH for small bowel and small bowel - PTV. C) Small bowel DVH results for all cases. D) Small bowel-PTV DVH results for all cases.

Figure 1 and 2 shows examples of dose distribution for the preoperative and postoperative cases. Dosimetric data for PTV and OAR are recorded in table 3 and DVH results are shown in figures 3 and 4. All plans were normalized aiming to obtain V_95% _> 99% for the PTV. When we evaluated GTV (preoperative cases)-CTV (postoperative cases) DVH in Figure 3, we could observe that for all cases the dose distribution was homogeneous. Nevertheless, homogeneity (represented by D_5%_-D_95%_) inside the PTV could reach 12 and 18% for the two largest volumes (6198 and 4085 cc) of the preoperative group.

**Table 3:** Dosimetric results for PTV and OAR.

	Preoperative				Postoperative			
	
	MEAN	SD	MAX	MIN	MEAN	SD	MAX	MIN
**PTV**								
Volume [cc]	2318,5	2223,9	6198,0	348,0	698,3	216,6	933,0	463,0
D1% [%]	111,2	6,7	124,0	103,4	105,3	2,4	109,1	103,1
D95% [%]	99,3	2,3	103,9	96,8	97,5	0,4	98,2	97,3
D5% [Gy]	109,4	6,2	121,8	102,5	104,5	2,0	107,6	102,6
V107% [%]	25,6	30,9	90,0	0,0	2,1	3,6	8,3	0,0
V95% [%]	99,0	0,0	99,0	99,0	99,0	0,0	99,0	99,0
D5%-D95% [%]	10,1	4,1	17,9	5,7	6,9	1,7	9,4	5,3
CI 95%	1,1	0,1	1,2	1,1	1,2	0,0	1,3	1,2
**Spinal Cord**								
D1% [Gy]	28,1	12,6	40,0	1,9	32,6	4,4	39,2	28,1
Dmax [Gy]	32,0	13,6	44,0	3,1	35,0	4,8	41,0	30,0
**Kidney**								
Volume [cc]	149,9	60,1	173,8	105,2	171,6	31,6	209,6	139,0
V5 Gy [%]	21,5	23,3	55,0	0,0	3,1	2,6	7,3	0,0
Dmean [Gy]	3,5	1,9	5,2	1,4	2,9	0,5	3,8	2,5
D1% [Gy]	5,6	2,4	7,6	3,6	5,4	0,7	6,0	4,3
**Bowel**								
Volume [cc]	1421,3	729,7	2720,0	628,8	1494,9	533,7	2406,0	1105,0
V30Gy [%]	33,2	12,0	50,9	19,0	30,5	11,5	43,0	11,9
V40Gy [%]	22,4	9,1	35,4	10,6	15,6	9,6	28,9	1,7
D1% [Gy]	53,4	2,9	59,6	50,7	46,9	0,8	47,7	45,7
V Prescription dose [%]	12,2	9,2	28,0	2,8	8,7	7,7	21,0	0,0
**Bowel-PTV**								
Volume [cc]	1183,6	645,3	2392,0	481,4	1343,7	613,2	2205,0	825,2
V30Gy [%]	18,5	7,1	30,5	10,1	21,6	7,5	30,0	9,4
V40Gy [%]	7,5	4,4	14,1	1,3	4,7	3,3	8,0	0,0
V Prescription dose [%]	0,6	0,9	2,2	0,0	0,7	1,3	2,9	0,0

Concerning the OAR, the dose constraints initially required (Table 1) were largely respected. With regards to the bowel and bowel-PTV we presented the DVH results for all cases, showing the important variability of bowel volume from one case to another. V_40Gy _ranged from 66.6 cc to 962.8 cc for group A and from 18.7 cc to 695.3 cc for group B. Mean small bowel D_1% _was 53 ± 2.9 Gy, with a D_max _of 59 Gy in the portion included in the PTV for the largest tumor. The volume of small bowel-PTV receiving the prescribed dose was always below 3 cc.

Dose constraints were largely respected for the kidney and the spinal cord.

### Early clinical practice

Treated patients were 29 and 47 years old respectively, and were diagnosed with a liposarcoma at the histological examination. They did not present any comorbidity factors. The treatment strategy was approved by a pluridisciplinar committee. PTV volumes were 933 and 463 cc, respectively. They underwent surgery combined to IORT at a single dose of 15 Gy delivered by an 80 mm diameter collimator, and then received postoperative radiotherapy at a dose of 45 Gy in 25 fractions.

Acute toxicity was evaluated according to the Common Toxicology Criteria grading system (CTC V.03). Both patients showed G1 nausea-vomiting. Pain and neuropathy was G0 and no patient presented any skin reactions or weight loss.

## Discussion

IMRT for retroperitoneal sarcoma has already been studied and implemented to clinical practice by some teams. Dose constraints criteria of those series are shown in Table 1. On the one hand, IMRT has proved a significant improvement of the PTV coverage when compared to 3DCRT, achieving a better protection of OAR, specially the small bowel (V_30 _43.1 ± 20.6% with IMRT vs 63.5 ± 25.2% with 3DCRT) [14]. On the other hand, the problem of IMRT for the treatment of important volumes, as some retroperitoneal sarcomas, is the difficulty to achieve a homogeneous dose distribution inside the PTV, which is translated in hotspots around OAR. To palliate this technical problem it is sometimes necessary to multiply fields or adding segments, that inevitably prolongs treatment delivery time. This implies the increased possibility of positioning error and the necessity of a trustworthy repositioning system, that is sometimes very inconfortable for the patient [16]. Knowing that the highest risk of local relapse is limited to the contact region between the tumor and the posterior abdominal wall, Bossi et al [13] proposed a new IMRT strategy in which the CTV was limited to this area, reducing the volume of the target in an attempt to decrease toxicity. IMRT plans were compared to 3DCRT and showed a significant better sparing with IMRT of the contralateral kidney. No significant advantage for small bowel was observed with IMRT in their study where they defined the CTV as a part of the whole GTV. Additionally, the presence of the tumor shifted small bowel outside of the PTV.

Many authors reported for other tumor sites dosimetric plans at least similar for RapidArc when compared to IMRT with a static gantry position [18-23]. RapidArc was implemented since 2008 in our institution in a daily practice for several localisations. Therefore we decided to evaluate this innovative technique for the treatment of retroperitoneal sarcomas.

We found in the frame of our dosimetric study better DVH results than those expected at the initial planning time taking into account that we studied very large volumes (Table 3). Our choice regarding the normalization method was specific for this localisation. We initially decided to cover 99% of the PTV by 95% of the prescribed dose. This resulted in a better dose coverage in the edge of the volume, but compromised homogeneity, particularly for the largest preoperative case, where we obtained a maximal dose of 124% inside the PTV. This hotspot wouldn't have been observed if we had covered 95% of the volume by 95% of the dose. Nevertheless, we may wonder whether the presence of these hotspots inside the PTV is really problematic knowing that this lesion will be removed.

Regarding the organs at risk, small bowel DVH showed that V_30Gy _and V_40Gy _results were better than initially required for both groups. Hotspots in the small bowel were systematically in the portion included in the PTV for the biggest case. The portion of bowel - PTV irradiated above the prescribed dose was always very limited (< 3cc).

To allow reproducible correlation between the volume of small bowel receiving a dose range and toxicity, DVH data were expressed in cc. Some authors showed that a V_30Gy _> 450 cc was correlated to a significant higher acute gastro-intestinal (GI) toxicity [24] and that when small bowel - PTV V_40Gy _exceeded 200 cc, there was a 10% probability to develop G2-3 acute GI toxicity [25]. Tzeng [26] treated 16 patients with retroperitoneal sarcoma at a dose of 45 Gy in 25 fractions using IMRT with a boost of 12.5 Gy to the areas at theoretical risk of positive margin after resection. The only patient showing G3 GI toxicity had received 54 Gy to more than 20 cc of small bowel, recommending that this constraint should be respected. Our small bowel DVH results always remained under these levels.

Kidney tolerance doses to whole organ irradiation DT5/5 and 50/5, are 23 and 28 Gy, respectively [27]. It has been reported that in the absence of concomitant chemotherapy or latent nephropathy, doses under 15 Gy are not likely to provoke radiation-induced nephropathy [28]. Another important concept is that kidney consists of multiple independent functional structures very sensitive to radiation. For this reason, despite the problem of total dose, there is the problem of quantity of irradiated volume even at low doses. May et al [29] showed that the percentage of bilateral renal volume receiving at least 10 Gy and the mean kidney dose were significant predictors of subsequent G2 renal complications (p = 0.017 and p = 0.0095 respectively).

In our study respectively mean and maximal doses received by the contralateral kidney were 3.45 Gy and 7.6 Gy for the preoperative and 2.94 Gy and 6 Gy for the postoperative plans, which is much lower than accepted doses. One could be worried about the respiration-induced motion of the kidneys making uncertain the doses received. Some authors studied this phenomenon showing a maximal movement of kidneys in cephalo-caudal direction, with displacements varying around 16 ± 8 mm [30,31] justifying the PRV of 3 cm that we created around this structure to allow respect of dose constraints. Furthermore, as those patients will be monorenal in most of the cases, we recommend the prescription of a pre-treatment renal scintigraphy to asses the functionality of the remaining kidney.

Concerning the dose for retroperitoneal sarcomas, limitation of dose prescription was assessed by the tolerance of the organs at risk. Our results open the question of dose escalation and will be the object of further studies.

Another important point is the reduction achieved in delivery time, which is a major advantage of RapidArc. Even if static gantry IMRT allows acceptable dose distribution, the average fraction time is about 20 minutes [13,20]. Shorter treatment time will reduce the likelihood of intrafraction baseline shifts in PTV and organs at risk position. Taking into account that those patients are painful in most of the cases because of the psoas invasion and have big difficulties to stay laying on the accelerator table, RapidArc technology offers a solution improving treatment comfort and decreasing the possibility of set-up errors.

Even if the available evidence from retrospective studies and prospective non randomized trials strongly suggests that conventional preoperative radiation is better tolerated, we treated using RapidArc technology two patients of the postoperative group with excellent clinical tolerance.

## Conclusions

RapidArc for retroperitoneal sarcomas achieved acceptable dosimetric results in preoperative or postoperative setting, even for large volumes. The two first treated patients presented a good tolerability. Currently, we are continuing to treat patients with this technique offering a rapid and safe procedure. Longer follow-up is warranted to assess long-term toxicity and local control.

## Competing interests

The authors declare that they have no competing interests.

## Authors' contributions

CLLM, PF and FQ designed and coordinated the study. Patient accrual and clinical data collection was done by CLLM and FQ. Data analysis, physics data and treatment planning data collection was done by PF and CLLM. CLLM prepared the manuscript. DA and PF revised critically for important intellectual content. All authors read and approved the final manuscript.

## References

[B1] Karakousis CP Velez AF Gerstenbluth R Driscoll DL Resectability and survival in retroperitoneal sarcomas Ann Surg Oncol 1996 3 2 150 8 10.1007/BF02305794 8646515

[B2] Lewis JJ Leung D Woodruff JM Brennan MF Retroperitoneal soft-tissue sarcoma: analysis of 500 patients treated and followed at a single institution Ann Surg 1998 228 3 355 65 10.1097/00000658-199809000-00008 9742918 PMC1191491

[B3] Raut CP Pisters PW Retroperitoneal sarcomas: Combined modality treatment approaches J Surg Oncol 2006 94 1 81 7 10.1002/jso.20543 16788949

[B4] Catton CN O'Sullivan B Kotwall C Cummings B Hao Y Fornasier V Outcome and prognosis in retroperitoneal soft tissue sarcoma Int J Rad Oncol Biol Phys 1994 29 5 1005 10 10.1016/0360-3016(94)90395-6 8083069

[B5] Gilbeau L Kantor G Lagarde P Thomas L Kind M Richaud P Coindre JM Bonichon F Bui BN Surgical resection and radiotherapy for primary retroperitoneal soft tissue sarcoma Radiother and Oncol 2002 65 3 137 43 10.1016/S0167-8140(02)00283-9 12464441

[B6] Stoeckle E Coindre JM Bonvalot S Kantor G Terrier P Bonichon F Nguyen Bui B French Federation of Cancer Centers Sarcoma Group Prognostic factors in retroperitoneal sarcoma: a multivariate analysis of a series of 165 patients of the French Cancer Center Federation Sarcoma Group Cancer 2001 92 2 359 68 10.1002/1097-0142(20010715)92:2<359::AID-CNCR1331>3.0.CO;2-Y 11466691

[B7] kinsella TJ Syndelar WF Lack E Glatstein E Rosenberg SA Preliminary results of a randomized study of adjuvant radiation therapy in resectable adult retroperitoneal soft tissue sarcomas J Clin Oncol 1988 6 1 18 25 3275748 10.1200/JCO.1988.6.1.18

[B8] Sindelar WF Kinsella TJ Chen PW DeLaney TF Tepper JE Rosenberg SA Glatstein E Intraoperative radiotherapy in retroperitoneal sarcomas. Final results of a prospective, randomized, clinical trial Arch Surg 1993 128 4 402 10 8457152 10.1001/archsurg.1993.01420160040005

[B9] Caudle AS Tepper JE Calvo BF Meyers MO Goyal LK Cance WG Kim HJ Complications associated with neoadjuvant radiotherapy in the multidisciplinary treatment of retroperitoneal sarcomas Ann Surg Oncol 2007 14 2 577 82 10.1245/s10434-006-9248-9 17119868

[B10] Jones JJ Catton CN O'Sullivan B Couture J Heisler RL Kandle RA Swallow CJ Initial results of a trial of preoperative external-beam radiation therapy and postoperative brachytherapy for retroperitoneal sarcoma Ann Surg Oncol 2002 9 4 346 54 10.1007/BF02573869 11986186

[B11] Gieschen Hl Spiro IJ Suit HD Ott MJ Rattner DW Ancukiewicz M Willet CG Long-term results of intraoperative electron beam radiotherapy for primary and recurrent retroperitoneal soft tissue sarcoma Int J Radiat Oncol Biol Phys 2001 50 1 127 31 11316555 10.1016/s0360-3016(00)01589-3

[B12] Petersen IA Haddock MG Donohue JH Nagorney DM Grill JP Sargent DJ Gunderson LL Use of intraoperative electron beam radiotherapy in the management of retroperitoneal soft tissue sarcoma Int J Radiat Oncol Biol Phys 2002 52 2 469 75 11872294 10.1016/s0360-3016(01)02595-0

[B13] Bossi A De Wever I Van Limbergen E Vanstraelen B Intensity modulated radiation-therapy for preoperative posterior wall irradiation of retroperitoneal liposarcomas Int J Radiat Oncol Biol Phys 2007 67 1 164 70 Erratum in: Int J Radiat Oncol Biol Phys. 2007,**68(1):**317. Dosage error in article text 17084556 10.1016/j.ijrobp.2006.08.023

[B14] Koshy M Landry JC Lawson JD Staley CA Esiashvili N Howell R Ghavidel S Davis LW Intensity modulated radiation therapy for retroperitoneal sarcoma: a case for dose escalation and organ at risk toxicity reduction Sarcoma 2003 7 3-4 137 48 10.1080/13577140310001644751 18521378 PMC2395528

[B15] Musat E Kantor G Caron J Lagarde P Laharie H Stoeckle E Angles J Gilbeau L Bui BN Comparison of intensity-modulated postoperative radiotherapy with conventional postoperative conformal radiotherapy for retroperitoneal sarcoma Cancer Radiother 2004 8 4 255 61 15450519 10.1016/j.canrad.2004.05.001

[B16] Hong L Alektiar K Chui C LoSasso T Hunt M Spirou S Yang J Amols H Ling C Fuks Z Leibel S IMRT of large fields: whole-abdomen irradiation Int j Radiat Oncol Biol Phys 2002 54 1 278 89 12183002 10.1016/s0360-3016(02)02921-8

[B17] Nicolini G Vanetti E Clivio A Fogliata A Korreman S Bocanek J Cozzi L The GLAas algorithm for portal dosimetry and quality assurance of RapidArc and intensity modulated rotational therapy Radiation Oncology 2008 3 24 10.1186/1748-717X-3-24 18782447 PMC2553075

[B18] Cozzi L Dinshaw KA Shrivastava SK Mahantshetty U Engineer R Deshpande DD Jamema SV Vanetti E Clivio A Nicolini G Foliata A A treatment planning study comparing volumetric arc modulation with RapidArc and fixed field IMRT for cervix uteri radiotherapy Radiother Oncol 2008 89 2 180 91 10.1016/j.radonc.2008.06.013 18692929

[B19] Palma D Vollans E James K Nakano S Moiseenko V Shaffer R McKenzie M Morris J Otto K Volumetric modulated arc therapy for delivery of prostate radiotherapy: comparison with intensity-modulated radiotherapy and three-dimensional conformal radiotherapy Int J Radiat Oncol Biol Phys 2008 72 4 996 1001 18455326 10.1016/j.ijrobp.2008.02.047

[B20] Alexander AS Wells D Berrang T Parsons C Mydin A Shaffer R Wong F Sayers D Otto K Volumetric Arc Therapy (VMAT) reduces treatment time compared to conventional IMRT (cIMRT) while mantaining similar plan quality in whole pelvic gynecologic radiotherapy Int J Radiat Oncol Biol Phys 2008 72 1 S366

[B21] Fogliata A Clivio A Nicolini G Vanetti E Cozzi L Intensity modulation with photons for benign intracranial tumours: a planning comparison of volumetric single arc, helical arc and fixed gantry techniques Radiother Oncol 2008 89 3 254 62 10.1016/j.radonc.2008.07.021 18760851

[B22] Clivio A Fogliata A Francetti-Pellanda A Nicolini G Vanetti E Wyttenbach R Cozzi L Volumetric-modulated arc radiotherapy for carcinomas of the anal canal: A treatment planning comparison with fixed field IMRT Radiother Oncol 2009 92 1 118 24 10.1016/j.radonc.2008.12.020 19181409

[B23] Vanetti E Clivio A Nicolini G Fogliata A Ghosh-Laskar S Agarwal JP Upreti RR Budrukkar A Murthy V Deshpande DD Shrivastava SK Dinshaw KA Cozzi L Volumetric modulated arc radiotherapy for carcinomas of the oro-pharynx and larynx: a treatment planning comparison with fixed field gantry Radiother Oncol 2009 92 1 111 7 10.1016/j.radonc.2008.12.008 19157609

[B24] Devisetty K Mell LK Salama JK Schomas DA Miller RC Jani AB Roeske JC Aydogan B Chumura SJ A multi-institutional acute gastrointestinal toxicity analysis of anal cancer patients treated with concurrent intensity-modulated radiation therapy (IMRT) and chemotherapy Radiother Oncol 2009 93 298 301 10.1016/j.radonc.2009.07.006 19717198

[B25] Fiorino C Alongi F Perna L Broggi S Cattaneo GM Cozzarini C Di Muzio N Fazio F Calandrino R Dose-volume relationships for acute bowel toxicity in patients treated with pelvic nodal irradiation for prostate cancer Int J Radiat Oncol Biol Phys 2009 75 1 29 35 19467803 10.1016/j.ijrobp.2008.10.086

[B26] Tzeng CW Fiveash JB Popple RA Arnoletti JP Russo SM Urist MM Bland KI Heslin MJ Preoperative radiation therapy with selective dose escalation to the margin at risk for retroperitoneal sarcoma Cancer 2006 107 2 371 9 10.1002/cncr.22005 16752414

[B27] Emami B Lyman J Brown A Coia L Goitein M Munzenrider JE Shank B Solin LJ Wesson M Tolerance of normal tissue to therapeutic irradiation Int J Radiat Oncol Biol Phys 1991 21 1 109 122 2032882 10.1016/0360-3016(91)90171-y

[B28] Cassady JR Clinical radiation nephropathy Int J Radiat Oncol Biol Phys 1995 31 5 1249 56 7713786 10.1016/0360-3016(94)00428-N

[B29] May KS Khushalani NI Chandrasekhar R Wilding GE Lyer RV Ma WW Flaherty L Russo RC Fakih M Kuvshinoff BW Gibbs JF Javle MM Yang Gy Analysis of clinical and dosimetric factors assocated with change in renal function in patients with gastrointestinal malignances after chemoradiation to the abdomen Int J Radiat Oncol Biol Phys 2010 76 4 1193 8 19540051 10.1016/j.ijrobp.2009.03.002

[B30] Ahmad NR Huq MS Corn BW Respiration-induced motion of the kidneys in whole abdominal radiotherapy Radiother Oncol 1997 42 1 87 90 10.1016/S0167-8140(96)01859-2 9132831

[B31] Bussels B Goethals L Feron M Bielen D Dymarkowski S Suetens P Haustermans K Respiration-induced movement of the upper abdominal organs: a pitfall for the tree-dimensional conformal radiation treatment of pancreatic cancer Radiother Oncol 2003 68 1 69 74 10.1016/S0167-8140(03)00133-6 12885454

